# Mutation in Rice *Abscisic Acid2* Results in Cell Death, Enhanced Disease-Resistance, Altered Seed Dormancy and Development

**DOI:** 10.3389/fpls.2018.00405

**Published:** 2018-03-28

**Authors:** Yongxiang Liao, Que Bai, Peizhou Xu, Tingkai Wu, Daiming Guo, Yongbin Peng, Hongyu Zhang, Xiaoshu Deng, Xiaoqiong Chen, Ming Luo, Asif Ali, Wenming Wang, Xianjun Wu

**Affiliations:** ^1^Rice Research Institute, Sichuan Agricultural University, Sichuan, China; ^2^Agriculture and Food, Commonwealth Scientific and Industrial Research Organization (CSIRO), Canberra, ACT, Australia

**Keywords:** abscisic acid, xanthoxin dehydrogenase, gibberellin, ABA/GA ratio, lesion mimic mutant, pre-harvest sprouting, defense responses, *Oryza sativa* L.

## Abstract

Lesion mimic mutants display spontaneous cell death, and thus are valuable for understanding the molecular mechanism of cell death and disease resistance. Although a lot of such mutants have been characterized in rice, the relationship between lesion formation and abscisic acid (ABA) synthesis pathway is not reported. In the present study, we identified a rice mutant, *lesion mimic mutant 9150* (*lmm9150*), exhibiting spontaneous cell death, pre-harvest sprouting, enhanced growth, and resistance to rice bacterial and blast diseases. Cell death in the mutant was accompanied with excessive accumulation of H_2_O_2_. Enhanced disease resistance was associated with cell death and upregulation of defense-related genes. Map-based cloning identified a G-to-A point mutation resulting in a D-to-N substitution at the amino acid position 110 of OsABA2 (LOC_Os03g59610) in *lmm9150*. Knock-out of *OsABA2* through CRISPR/Cas9 led to phenotypes similar to those of *lmm9150*. Consistent with the function of *OsABA2* in ABA biosynthesis, ABA level in the *lmm9150* mutant was significantly reduced. Moreover, exogenous application of ABA could rescue all the mutant phenotypes of *lmm9150*. Taken together, our data linked ABA deficiency to cell death and provided insight into the role of ABA in rice disease resistance.

## Introduction

Abscisic acid (ABA) is one of the multi-functional phytohormones that is involved in many essential physiological processes during growth and development in plants, such as seed maturation, seed desiccation, seed dormancy, germination, and stress-induced responses (Rock and Quatrano, 1995; Leung and Giraudat, 1998; Maia et al., 2014). In rice, ABA modulates seed dormancy mainly through the balance of ABA and GA (ABA/GA ratio) (Liu et al., 2010, 2014; Shu et al., 2013). In different plant–pathogen interaction systems, it is well-established that ABA negatively regulates plant defense. On one hand, ABA-deficient mutants, such as *abscisic acid2-1* (*aba2-1*) and *aba3-1* in *Arabidopsis, sitiens* (*sit*) in tomato, display enhanced resistance to *Golovinomyces cichoracearum* and *Erwinia chrysanthemi*, respectively (Asselbergh et al., 2008; Xiao et al., 2017). Conversely, ABA-increased mutants in *Arabidopsis* become more susceptible (Gao et al., 2016). On the other hand, exogenous application of ABA increases susceptibility to different pathogens in rice, *Arabidopsis* and tomato (Asselbergh et al., 2008; Xu et al., 2013; Xiao et al., 2017). ABA-treatment on rice plants leads to enhanced disease symptoms on both susceptible and resistant accessions (Koga et al., 2004; Jiang et al., 2010). However, to our knowledge there is no report of an ABA-deficient mutant associated with disease resistance in rice.

The biosynthesis of ABA has two pathways: (1) the direct pathway *via* mevalonate pathway in Fungi (Hirai et al., 2000; Inomata et al., 2004) and (2) the indirect pathway *via* carotenoid pathway that is the main biosynthesis pathway in higher plants. The indirect pathway contains three stages (Milborrow, 2001; Schwartz et al., 2003). First, zeaxanthin is synthesized by zeaxanthin epoxidase (ZEP) in plastids (Marin et al., 1996; Agrawal et al., 2001). Second, zeaxanthin is converted into 9-*cis*-violaxanthin and 9-*cis*-neoxanthin by neoxanthin synthase that is encoded by *ATABA4* (North et al., 2007). They are dissociated to xanthoxin (Xan) by *9-cis-epoxycarotenoid dioxygenase* (*NCED*) (Iuchi et al., 2001; Riahi et al., 2013; Zhang et al., 2014). Third, Xan is transferred to the cytosol and converted to abscisyl aldehyde (ABAld) by *xanthoxin dehydrogenase* (*XanDH*) (Miguel et al., 2002; Endo et al., 2014). Then, ABAld is catalyzed into ABA by *Abscisic Aldehyde Oxidase* (*AAO*) (Seo et al., 2000).

XanDH belongs to a short-chain dehydrogenase/reductase (SDR) superfamily that is encoded by *ABA2*. *AtABA2* is a single copy gene in *Arabidopsis thaliana* and is constitutively expressed. Expression of AtABA2 activates the glucose signal, antagonizes the ethylene signal and promotes the synthesis of ABA (Cheng et al., 2002; Miguel et al., 2002). *ZmABA2* is the homolog of *AtABA2* in maize. ZmABA2 interacts with ZmMPK5 and coordinately regulates the ABA level in maize (Ma et al., 2016). *OsABA2*, a rice *ABA2* homolog, could restore the *Arabidopsis ataba2* mutant phenotypes (Endo et al., 2014), indicating the conservation of ABA biosynthesis function of *OsABA2*. However, the phenotypic changes in rice resulted from mutation in *OsABA2* has not been described.

Previous studies have described mutants that have disrupted functions of genes in ABA biosynthetic pathway in different plants, such as *sitiens* (*sit*) and *notalilis* (*not*) in tomato (Burbidge et al., 1999; Okamoto et al., 2002), *aba1, aba2*, and *aba3* in *Arabidopsis* (Léon-Kloosterziel et al., 1996; Schwartz et al., 1997a; Xiong et al., 2002) and *pre-harvest sprouting* (*phs*) and *viviparous* (*vp*) mutants (*vp10, vp13*, and *vp14*) in maize (Tan and Mccarty, 1997; Schwartz et al., 1997b, 2003). The grains of those *phs* mutants easily germinate in the ear or panicle before harvest under wet conditions. In rice, the *osaba1* mutant has a mutation in the gene encoding zeaxanthin epoxidase that involves in ABA synthesis in rice (Agrawal et al., 2001). The genes of *phytoene desaturase* (*OsPSD*), ζ*-carotene desaturase* (*OsZDS*), *carotenoid isomerase* (*OsCRTISO*), and *lycopene* β*-cyclase* (*β-OsLCY*) encode essential enzymes in different steps of the carotenoid synthetic pathway and the disruption of carotenoid biosynthesis leads to PHS trait (Fang et al., 2008). How other genes involve in ABA biosynthesis modulate pre-harvest sprouting remains to be tested.

The lesion mimic mutants (LMMs) display cell death, similar to hypersensitive response (HR), and are highly valuable in the investigation of the molecular mechanism of programmed cell death and defense responses (Greenberg, 1997; Moeder and Yoshioka, 2008; Tamiru et al., 2016). A number of LMMs have been isolated and characterized in *Arabidopsis* (Cao et al., 1994), maize (Hu et al., 1998), barley (Persson et al., 2009), and rice (Kiyosawa, 1970; Liu et al., 2017). In rice, more than 15 LMM-related genes have been isolated and characterized, which encode proteins of distinct functions, such as OsCUL3a that is the prominent component of cullin 3-based RING E3 ubiquitin ligases (Liu et al., 2017), an AAA-type ATPase (Fekih et al., 2015; Zhu et al., 2016), an eEF1A-like protein (Wang et al., 2017), a mitogen-activated protein kinase kinase kinase (Wang S. et al., 2015), a heat stress transcription factor protein (Utako et al., 2002), a cytochrome P450 monooxygenase (Tadashi et al., 2010), an U-box/armadillo repeat protein (Zeng et al., 2004), a clathrin-associated adaptor protein comlex1 (Qiao et al., 2009), a splicing factor 3b subunit 3 protein (Chen et al., 2012), and a thylakoid-bound protein (Li et al., 2010). Therefore, the molecular mechanism of lesion mimic formation in plant is regulated by a complicated regulatory network. Although a variety of LMM-related genes have been identified, whether ABA synthesis pathway is involved in lesion formation and how ABA modulates disease response in rice remains to be answered.

Here, a new rice *lesion mimic mutant 9150*, named *lmm9150*, was isolated and characterized. The *lmm9150* mutant exhibited spontaneous cell death on leaves, pre-harvest sprouting under field condition, and enhanced growth of leaves and stems. The lesions in *lmm9150* were associated with typical defense responses, such as accumulation of H_2_O_2_ and enhanced resistance to bacterial blight and rice blast diseases. Using a map-based cloning approach, we detected a point mutation in *LOC_Os03g59610*, which encodes OsABA2, a short-chain alcohol dehydrogenase. Knocking-out of *OsABA2* with CRISPR/Cas9 led to lesion mimic spots, enhanced disease resistance and growth, and PHS traits. Consistence with the function of OsABA2 in ABA synthesis, the lesion mimic spots and other phenotypes of *lmm9150* could be rescued by exogenous application of ABA. Therefore, our results provide further insights into the molecular function of *OsABA2* in lesion mimic formation, disease-resistance, seed dormancy, and development in rice.

## Materials and Methods

### Plant Materials and Growth Conditions

The *lmm9150* mutant was generated by ethyl methane sulfonate (EMS) treatment of a Chinese *indica* cultivar Yixiang1B, which is one of elite backbone parents in hybrid rice breeding programs in China. The wild type (WT) Yixiang1B and *lmm9150* were grown, respectively, in the paddy field in Chengdu city (N30.67°E104.06°), Sichuan Province and in Lingshui county (N18.47°E110.04°), Hainan Province, China.

### Histochemical Analysis

Leaves from the *lmm9150* mutant, with obvious lesion mimic spots, at the seedling stage and the WT at the same growth stage were collected for histochemical analysis. Trypan blue staining was performed to detect cell death as previously described (Zhu et al., 2016). In brief, samples were submerged in lactic acid-phenol-trypan blue solution (2.5 mg/ml trypan blue, 25% (w/v) lactic acid, 23% water-saturated phenol, and 25% glycerol in H_2_O) and kept at room temperature for 6–12 h, followed by de-staining with solution containing 30% (w/v) chloral hydrate for 5 days. Then, the samples were equilibrated with 50% glycerol for 1 day for taking photos. For detection of hydrogen peroxide (H_2_O_2_) accumulation, the 3,3′-diaminobenzidine (DAB) staining was performed as described previously (Zhu et al., 2016).

### Chlorophyll Content Measurement

Leaves were soaked in 20 ml of 95% alcohol for 48 h in dark, until the leaves became colorless. The absorbance values were examined by a spectrophotometer at 665 and 649 nm. Then, the content of chlorophylls was calculated as follows:

Chlorophyll (Chl) a absorbance value = 13.95 × OD_665_-6.88 × OD_649_
Chlorophyll (Chl) b absorbance value = 24.96 × OD_649_-7.32 × OD_665_
Chlorophyll content (mg/g) = (C × V × D)/1000 × W

C: chl a or chl b absorbance value; V: volume of extracting solution; D: dilution index; W: weight of sample.

Chlorophyll content was measured in three biological repeats. Statistical analysis was performed using Student’s *t*-test.

### Inoculation of Pathogens and Disease Resistance Assay

The resistance assay to bacterial blight disease was performed as described previously. In brief, 70-days-old (at tillering stage) plants of the mutant *lmm9150* and the WT were used for inoculation of the bacterial pathogen *Xanthomonas oryzae* pv. *oryzae* (*Xoo*). The *Xoo* strains, P6, P3, 8248, and X004, which are compatible with the WT, were used for inoculation. *Xoo* bacterial suspensions with 0.5 of OD_600_ were used to inoculate by using the scissors-dip method (Zuo et al., 2014). Disease lesion lengths were measured at 15 days post-inoculation (dpi).

For resistance assay to rice blast disease, 60-days-old (at tillering stage) plants of the *lmm9150* mutant and the WT were used for inoculation with the fungal pathogen *Magnaporthe oryzae* (*M*. *oryzae*). The *M*. *oryzae* strains, ZhongI, and Tetep, which are compatible with the WT, were used for inoculation following a previous report (Park et al., 2012). The disease lesions were observed at 5 dpi.

### DNA Extraction and PCR, RNA Extraction, and qRT-PCR

Genomic DNA was extracted from leaves using the cetyltrimethylammonium bromide (CTAB) method (Murray et al., 1980). The PCR mixture was mixed with 2 μl DNA (10–50 ng/μl), 2 μl primers (10 μmol/μl), 0.3 μl dNTPs (10 mmol/L), 0.2 μl Taq (5 U/μl), and 13.5 μl H_2_O. The running procedure of PCR was performed as the following: pre-denaturation at 95°C for 5 min followed by 30 cycles of denaturation at 95°C for 30 s, annealing at 56°C for 30 s, extension at 72°C for 1 min, with a final extension at 72°C for 10 min. The PCR products were separated by electrophoresis in a 3% agarose gels, stained with ethidium bromide (EB) and photographed.

Total RNA was extracted using Trizol (Invitrogen, Carlsbad, CA, United States) following the procedures of the manufacturer. The mRNA was digested with DNase I according to the manufacturer’s instructions (Invitrogen, Carlsbad, CA, United States) and was subjected to reverse transcription to synthesize first-stand cDNA. Oligo (dT) was used as primer and SuperScript II (Invitrogen, Carlsbad, CA, United States) was used as reverse transcription enzyme.

The qRT-PCR was performed using a Bio-Rad CFX96 Real-Time System coupled to a C1000 Thermal Cycler (Bio-Rad, Hercules, CA, United States). The housekeeping gene *Ubiquitin5* (*Ubq5*) was used as the internal control. The sequences of the primers were listed in **Supplementary Table S1**.

### Genetic Analysis and Map-Based Cloning

Two F_1_ and two F_2_ populations derived from the crosses of Yixiang1B × *lmm9150* and *lmm9150* × Yixiang1B were used for genetic analysis. The F_2_ population derived from the cross of 02428 ×*lmm9150* was used for mapping of the mutant gene. For the bulk segregation analysis (BSA) (Michelmore et al., 1991), equal amount of leaf blades from 10 F_2_ plants with the lesion mimic phenotype and 10 F_2_ plants with the WT phenotype were collected for DNA extraction to construct the mutant and the WT DNA pools, respectively. The physical linkage map was then constructed using molecular markers nearby the *lmm9150* gene.

The SSR primers were synthesized according to the information of Gramene database^1^. InDel markers were developed based on the alignment results of the reference 93–11, an *indica* rice^2^ and the Nipponbare, a *japonica* rice^2^ genome sequence at the candidate region. Primers were designed using Primer3 web version4.0.0^3^. The specificity of each primer in the rice genome was confirmed by BLAST^4^ and PCR analysis. The sequences of SSR and InDel markers were listed in **Supplementary Table S2**.

For analysis of the PCR products, amplified products were separated by electrophoresis at 3.0% agarose gel in 0.5 × TBE buffer, and visualized and photographed under UV light.

For whole-genome re-sequencing, the *lmm9150* was backcrossed with the WT and self-crossed to generate BC_1_F_2_ population. The equal total DNA of 20 BC_1_F_2_ plants with lesion mimic spots, was mixed and re-sequenced at Novogene Corporation (Beijing, China). At the same time, the WT genome DNA was also sequenced as control.

### Sequence Analysis

Gene prediction was performed using the Rice Genome Annotation Project database^5^. Sequence alignments were performed using the software DNAMAN Version 6.0. Alignments of amino acid were performed using the software Clustax2.

### Construction of Knockout Lines by CRISPR/Cas9

Vector construction was performed to knockout the candidate gene as previously described (Ma et al., 2015). In brief, two targets (**Supplementary Table S3**), named lmm9150-Y and lmm9150-B, were designed in the coding sequence of conservative region. The two target sequences were amplified by PCR. Using endonuclease, Eco311 and T4 ligase, the lmm9150-Y was inserted into the pBWA (V) H-cas9i2 and the lmm9150-B was inserted into the pBWD (LB) DNAi. Finally, Pbwa (V) H-cas9i2-lmm9150-Y and pBWD (LB) DNAi-lmm9150-B were assembled into the final vector pBWA (V) H-cas9i2-lmm9150 using endonuclease SapI and T4 ligase. The constructs were verified by sequencing and then introduced into the WT by *Agrobacterium*-mediated transformation as described previously (Jeon et al., 2000). The sequences near the editing region were verified by extracting genomic DNA from T1 transgenic plants (sequencing primer in **Supplementary Table S3**).

### ABA, GA, and Water Loss Assay

Determination of ABA and GA content was carried out using enzyme-linked immunosorbent assay (ELISA) method as previously described (Teng et al., 2006). To test the water-loss rate, seedlings were grown in a paddy field and leaves (300 mg from 10 seedlings) of 40-days-old seedlings were kept on dish at room temperature (28°C). The weight of samples was measured at 20 min interval until 120 min.

### Germination Assay

Seeds from the *lmm9150* mutant and the WT at 30 days after flowering were used for germination test. Seeds were cultured in a growth chamber at 30°C,14 h light and 24°C,10 h dark cycle for 8 days.

## Results

### Isolation and Characterization of the *lmm9150* Mutant

**FIGURE 1:**
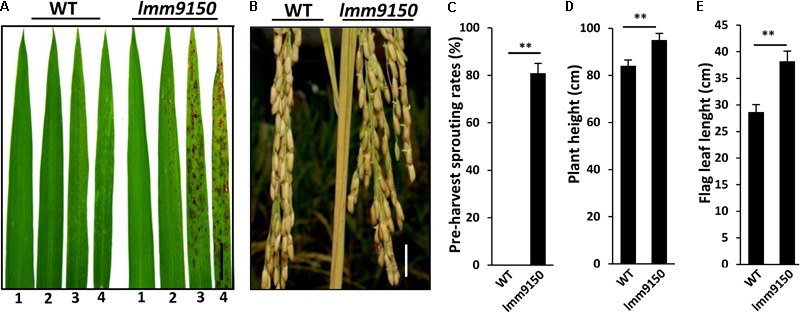
Phenotypic characterizations of *lmm9150* mutant. **(A)** Comparison of four leaves between wild type (WT) and *lmm9150* mutant shows the lesion mimic spots of *lmm9150* at seedling stage. The numbers 1, 2, 3, and 4 represent the first top leaf, the second top leaf, the third top leaf, and the fourth top leaf, respectively. **(B)** The *lmm9150* displays pre-harvest sprouting at yellow mature stage in the paddy field. **(C–E)** Quantification analyses on pre-harvest sprouting rates **(C)**, plant height **(D)**, and flag leaf length **(E)**, respectively. Data were obtained from 10 main panicles of plants **(C)**, 10 plants **(D)**, and 10 flag leaves of main panicles **(E)**. Statistical analysis was performed using Student’s *t*-test, ^∗∗^ indicates *P* < 0.01. Scale bar: 2 cm in **(A,B)**.

To explore the molecular connection of lesion mimic and disease resistance in rice, we carried out an extensive forward genetic screening for lesion mimic mutants from an EMS-mutagenized population of *indica* cultivar Yixiang1B. Subsequently, more than 20 mutants were identified. One of these mutants, named *lmm9150*, was chosen for further characterization because of its multiple phenotypes in development. The 3-week-old seedlings of the mutant *lmm9150* begun to develop brown lesions on the top part of the third leaf in the paddy field in both Chengdu (N: 30.67°) and Hainan (N: 18.47°) (**Figure 1A**). At tillering stage, the apical part of older leaves also displayed lesion mimic spots (**Supplementary Figure S1A**). At mature stage, the spots emerged to the top part of flag leaf (**Supplementary Figure S1B**). In addition, *lmm9150* mutant displayed obvious pre-harvest sprouting at 30 days after flowering under natural condition (**Figures 1B,C**).

However, apart from plant height and the flag leaf of *lmm9150* that were significantly larger than WT (**Figures 1D,E**), the yield-related agronomic traits, including the number of tillers, seed-setting rate, grain weight per plant, and 1000-grains weight, had no significant difference from those of WT (**Supplementary Figures S1C–F**).

**FIGURE 2:**
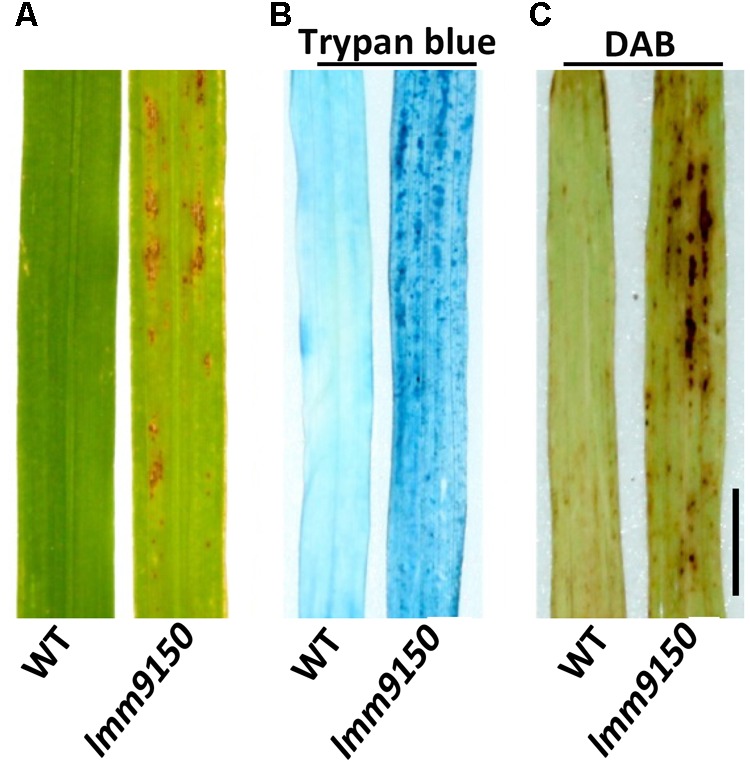
Observation of cell death in wild type (WT) and *lmm9150* mutant. **(A–C)** Representative leaf sections of WT and *lmm9150* mutant (MT) show lesions **(A)**, dead cells revealed by trypan blue staining **(B)**, and H_2_O_2_ accumulation revealed by 3,3′-diaminobenzidine (DAB) staining **(C)** in the mutant, respectively. The *lmm9150* mutant and WT leaf samples were collected at 30 days after sowing. Scale bar: 1 cm.

To characterize the lesions in *lmm9150*, we performed a trypan blue staining assay to examine cell death. Compared with WT, a lot of blue spots were observed on the leaf of *lmm9150* after staining (**Figure 2B**), indicating existence of dead cells. Because cell death is often associated with the production of reactive oxygen species (ROS) such as H_2_O_2_, we further examined the accumulation of H_2_O_2_ in *lmm9150* by DAB staining assay. Our data indicated that DAB-stained was observed in the mutant but not WT leaf blade, whereas there were rarely observed in the WT (**Figure 2C**).

**FIGURE 3:**
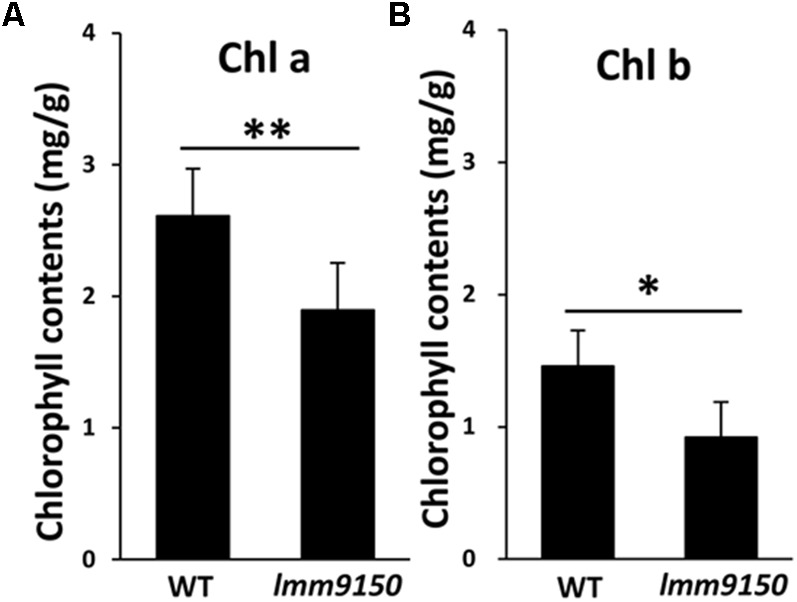
Comparison of the chlorophylls content between wild type (WT) and *lmm9150* mutant. **(A,B)** The content of Chlorophyll a (chl a) and chlorophyll b (chl b) shows reduction in *lmm9150* in comparison with WT. Mean and SD were obtained from three measurements. Statistical analysis was performed using Student’s *t*-test, ^∗^ and ^∗∗^ represents *P* < 0.05 and *P* < 0.01, respectively.

Next, we examined the content of chlorophylls. Small pieces from cognate positions of flag leaves of WT and *lmm9150* at flowering stage were collected for measurement of chlorophylls. The results showed that the content of both chlorophyll a and chlorophyll b in *lmm9150* was significantly lower than that of WT (**Figures 3A,B**), implying that the formation of lesion mimic spots might attributable to the decrease of photosynthesis pigments in the *lmm9150* mutant.

In summary, this mutant began to exhibit lesions as red brown spots from the top part of the third leaf at seedling stage, which eventually extended to the flag leaf at mature stage. Cell death was detected in the mutant, which was accompanied with excessive accumulation of H_2_O_2_. In addition, the mutant also exhibited higher plant height, longer flag leaf, and pre-harvest sprouting of seeds at mature stage.

### Enhanced Resistance to Bacterial Blight and Rice Blast Pathogens by *lmm9150*

**FIGURE 4:**
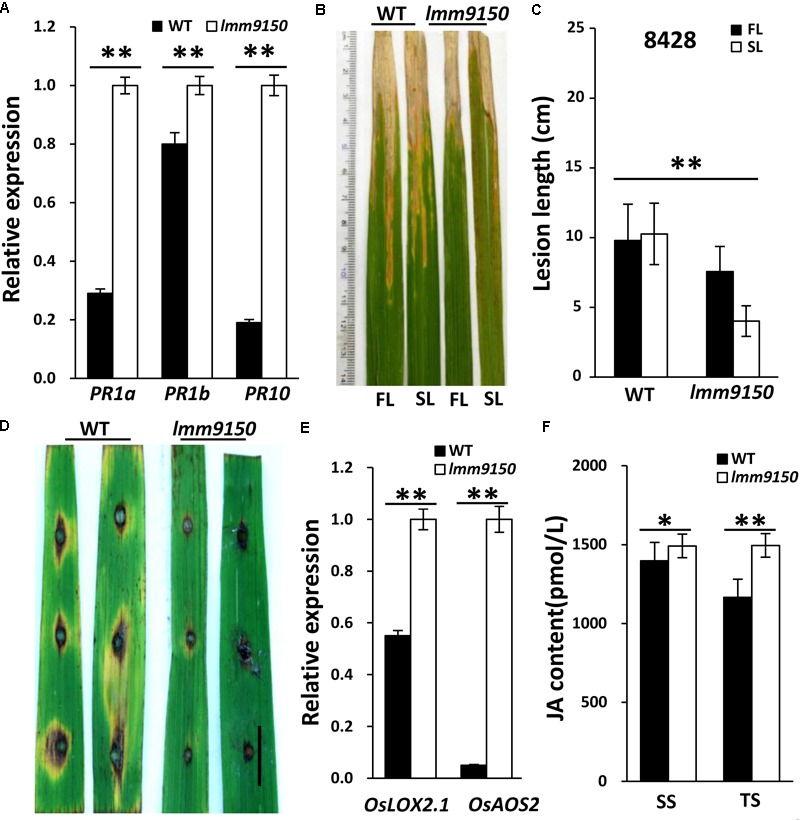
Determination of wild type (WT) and *lmm9150* plants resistance to bacterial blight and rice blast diseases. **(A)** Quantitative reverse-transcription PCR (qRT-PCR) data show the relative expression levels of pathogenesis-related (*PR*) genes in *lmm9150* and WT. Mean and SD were obtained from three measurements. **(B,C)** The disease lesion leaves of WT and *lmm9150* mutant were inoculated with *Xanthomonas oryzae* pv. *oryzae* (*Xoo*) strain, 8428, at 15 days post-inoculation. The image **(C)** shows that the disease lesions of WT are longer than that of *lmm9150* and the flag leaf disease lesions of *lmm9150* are longer than that of the second leaf of mutant. FL: the flag leaf; SL: the second leaf. Data were obtained from 20 leaves of main panicles. **(D)** The disease lesions of leaves of WT and *lmm9150* mutant were inoculated 5 days post-inoculation with *Magnaporthe* oryzae (*M*. oryzae) strain, ZhongI. The experiments were repeated twice with similar results. **(E)** Quantitative reverse-transcription PCR (qRT-PCR) data show the relative expression levels of jasmonic acid (JA) biosynthesis-related genes in *lmm9150* and WT. **(F)** Comparison of JA content in leaf of WT and *lmm9150* mutant. The content of hormone was tested by enzyme-linked immunosorbent assay (ELISA) method. Mean and SD were obtained from three measurements **(E,F)**. The housekeeping gene *Ubiquitin5* (*Ubq5*) was used as control. Statistical analysis was performed using Student’s *t*-test, ^∗^ and ^∗∗^ indicate *P* < 0.05 and *P* < 0.01, respectively. Scale bar: 1 cm in **(D)**.

Many lesion mimic mutants exhibit enhanced defense responses in plants (Wang J. et al., 2015; Zhu et al., 2016). We speculated that the *lmm9150* mutant may also become resistant to diseases in rice. To confirm this speculation, we first analyzed the expression of *Pathogenesis-Related* (*PR*) genes using quantitative reverse-transcription PCR (qRT-PCR), including *PR1a, PR1b, PR10* (Nahar et al., 2011; Sang et al., 2011). The results demonstrated that all the examined *PR* genes were significantly upregulated in *lmm9150* (**Figure 4A**), implying that *lmm9150* was a potential auto-immune mutant. Then, we tested disease resistance of *lmm9150* by inoculating *Xanthomonas oryzae* pv. *oryzae* (*Xoo*) and *Magnaporthe oryzae* at tillering stage, respectively. The results showed that the inoculated *lmm9150* leaves exhibited disease lesions obviously shorter than that of WT leaves at 15 days after inoculation of *Xoo* (**Figures 4B–E** and **Supplementary Figures S2A–C**), indicating enhanced resistance. Quantification of the lesion lengths on leaves revealed that the average disease lesion length in *lmm9150* was significantly shorter than that of WT. Intriguingly, the lesions on flag leaves were also significantly longer than those on the second leaves in *lmm9150* (**Figure 4C** and **Supplementary Figure S2D**). In addition, lesions caused by *Magnaporthe oryzae* on the *lmm9150* leaves were remarkably smaller than those of WT leaves (**Figure 4D** and **Supplementary Figure S2E**), indicating enhanced resistance to rice blast disease.

To explore the possible hormone pathway of defense responses activated in *lmm9150*, we examined the expression of jasmonic acid (JA) and salicylic acid (SA)-related genes. The *lipoxygenase2.1* (*OsLOX2.1*) and *allene oxide synthase2* (*OsAOS2*) are key genes of JA synthesis pathway (Liu et al., 2012). The *phytoalexin deficient4* (*OsPAD4*) and *lipase1* (*OsEDS1*) are key genes of SA signaling pathway (Pieterse et al., 2012). The results indicated that the expression of *OsLOX2.1* and *OsAOS2* was significantly higher in *lmm9150* than that in WT (**Figure 4E**), indicating increase of JA synthesis. In contrast, the expression of *OsPAD4* and *OsEDS1* had marginal difference between *lmm9150* and WT (**Supplementary Figure S2F**), implying that SA signaling pathway may not be activated in *lmm9150*. To address why SA signaling was not activated and confirm JA synthesis was increased, we examined SA level and JA level in leaf blades of WT and *lmm9150* at seedling stage and tillering stage. The results showed that JA level of *lmm9150* in leaf blades was significantly higher than that of WT at both stages, whereas SA level of *lmm9150* in leaf blades had no difference from that of WT (**Figure 4F** and **Supplementary Figure S2G**). These data indicated that JA biosynthesis is increased and thus JA-signaling pathway may be associated with the defense responses activated in *lmm9150*.

### Identification of *lmm9150* as a Mutant of *OsABA2*

The two F_1_ and two F_2_ populations derived from the crosses of Yixiang1B (WT) ×*lmm9150* and *lmm9150* × Yixiang1B were used for the genetic analysis. We found that all F_1_ plants were not observed lesion mimic spots on leaf blades and the segregation between WT and *lmm9150* in F_2_ population fitted 3:1 (**Supplementary Table S4**). Thus, *lmm9150* lesion mimic phenotype was controlled by a single recessive nuclear gene.

**FIGURE 5:**
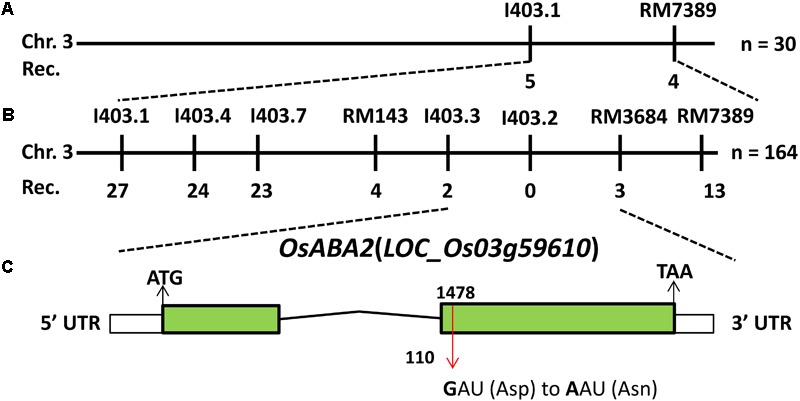
Positional cloning of *OsABA2*. **(A)** The *OsABA2* gene is located on chromosome 3 between InDel marker I403.1 and SSR marker RM7389. **(B)** The *OsABA2* gene is delimited to the region between I403.3 and RM3684 using 164 F_2_ mutant individuals. **(C)** The gene structure of *OsABA2.* Two exons and one intron are indicated by green rectangles and black lines, respectively; a G-to-A base substitution at the 1487th base was detected in the second exon (red arrow) leading to the change of Aspartic acid (Asp) to Asparagine (Asn) at the 110th amino acid.

To isolate the candidate gene that was responsible for the phenotypes of *lmm9150*, a mapping population was constructed by crossing *lmm9150* with a *japonica* cultivar 02428. Bulked segregation analysis (BSA) found that eight molecular markers on the end of chromosome 3 co-segregated with the mutant phenotypes of *lmm9150*. Linkage analysis showed that the mutant gene was mapped to a 488-kb interval between the InDel marker I403.3 and the SSR marker RM3684, co-segregated with I403.2 (**Figures 5A,B**). Next, the whole-genome was re-sequenced by using the DNA sample bulked from 20 BC_1_F_2_ individuals with the lesion mimic phenotype. The genomic DNA of the WT was also sequenced as a control. A single base mutation, which SNP index was one, was found by comparing sequences between the bulked mutants and WT in this interval. Because this SNP localized in the second exon of *LOC_Os03g59610*, we sequenced the gene in *lmm9150* and the WT. As expected, a G-to-A single base substitution was detected at the 1487th base in *lmm9150*, which resulted in a change from Aspartic acid (D) to Asparagine (N) at the 110th amino acid (**Figure 5C** and **Supplementary Figure S3**). These results indicated that *lmm9150* was likely arisen from the single base substitution in the *LOC_Os03g59610*. This gene encodes XanDH, which is orthologous of *ABA2* in *Arabidopsis* (Cheng et al., 2002; Miguel et al., 2002) and had been named *OsABA2* in rice (Endo et al., 2014).

Sequence alignment of *LOC_Os03g59610* between Nipponbare and Yixiang1B revealed a 9-bp deletion in the first exon resulting in deletion of 3 amino acid residue in Yixiang1 B (GenBank accession number: MG334011), which is the WT of *lmm9150* (**Supplementary Figure S4**).

**FIGURE 6:**
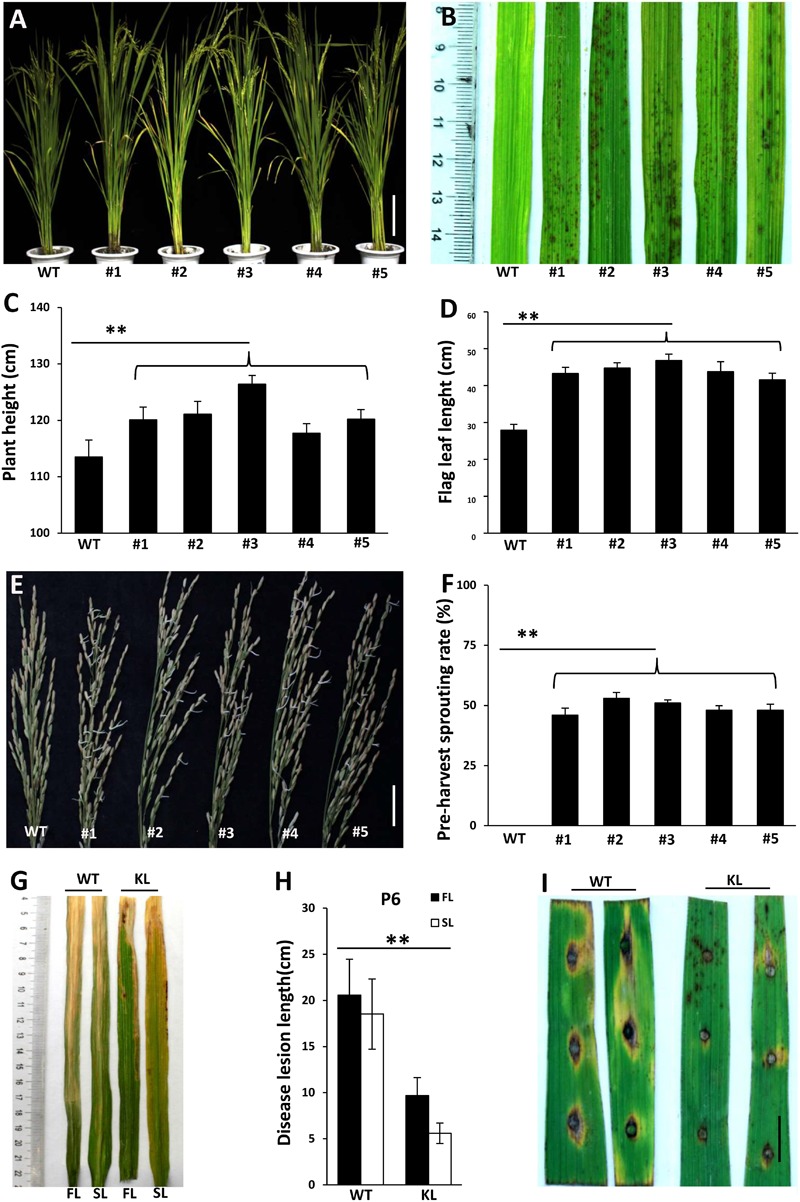
Phenotypic characterizations of T1 knockout lines. **(A)** The plant of the wild type (WT) and 5 T1 knockout lines. The mutations of #1, #3, #4, and #5 knockout lines are deletions. The mutation of #2 knockout line is insertion. **(B)** 5 knockout lines displays lesion mimic spots on leaves at tillering stage. **(C)** Comparison of plant height between WT and 5 knockout lines. **(D)** Comparison of flag leaf length between WT and 5 knockout lines. **(E)** The panicles for 30 days after flowering were collected and cultivated 3 days, at 28°C. All knockout lines display pre-harvest spouting. **(F)** Comparison of the pre-harvest sprouting rates between WT and 5 knockout lines 30 days after flowering under natural condition. **(G,H)** The disease lesion of leaves of WT and #3 knockout line were inoculated with *Xanthomonas oryzae* pv. *oryzae* (*Xoo*) strain, P6, at 15 days post-inoculation. KL: knockout lines; FL: the flag leaf; SL: the second leaf. Data were obtained from 10 plants **(C)**, 10 flag leaves of main panicles **(D)**, 15 main panicles **(F)**, and 20 leaves of main panicles **(H)**. **(I)** The disease lesions of leaves of WT and #3 knockout line was inoculated 5 days post-inoculation with *Magnaporthe oryzae* (*M. oryzae*) strain, ZhongI. The experiments were repeated twice with similar results. Scale bar: 15 cm in **(A)**, 5 cm in **(E)**, 1 cm in **(I)**. Statistical analysis was performed using Student’s *t*-test, ^∗∗^ indicates *P* < 0.01.

To confirm that the phenotypes of *lmm9150* were specifically associated with a mutation in *OsABA2*, we performed a knockout experiment in the WT background by using CRISPR/Cas9 editing. Five transgenic lines with either deletions or insertions in *OsABA2* were obtained (**Figure 6A** and **Supplementary Figure S5**). All of these transgenic lines displayed lesion mimic spots on leaves (**Figure 6B**). Furthermore, gene-edited lines showed higher plant heights (**Figure 6C**), longer flag leaves (**Figure 6D**), pre-harvest sprouting phenotype (**Figures 6E,F**), and enhanced resistance to bacterial blight and rice blast diseases (**Figures 6G–I** and **Supplementary Figures S6A–G**). Taken together, these data confirmed that the loss-of-function of *OsABA2* led to the *lmm9150* phenotypes.

### The Function of *OsABA2* in the Formation of Lesion Mimic Spots, Plant Development, and Seed Dormancy

**FIGURE 7:**
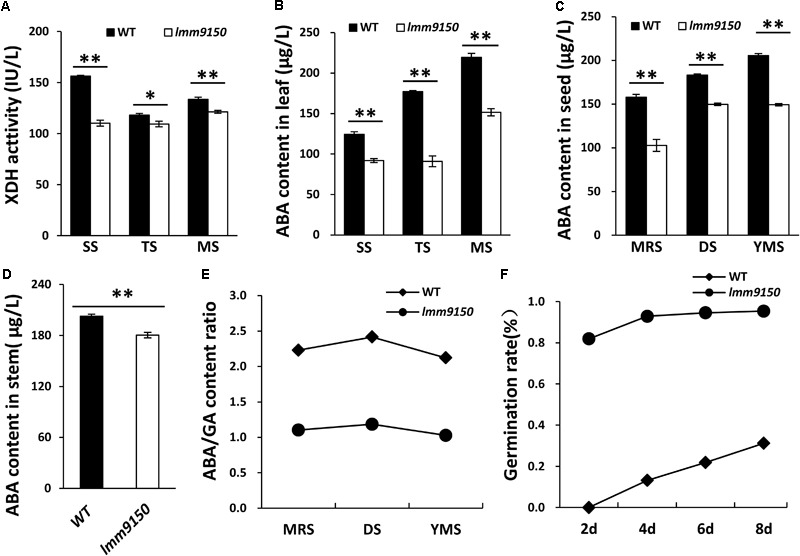
Comparison of xanthoxin dehydrogenase (XanDH) activity and abscisic acid (ABA) contents between wild type (WT) and *lmm9150* mutant. **(A)** Changes of XanDH activity in the leaf of WT and the *lmm9150* mutant. SS: seedling stage; TS: tillering stage; MS: mature stage. **(B–D)** Comparison of ABA contents in the leaves **(B)**, seeds **(C)**, and stems **(D)** between WT and the *lmm9150* mutant at different developmental stages. Hormone content was tested using enzyme-linked immunosorbent assay (ELISA) method. MRS: milk ripe stage; DS: dough stage; YMS: yellow mature stage. **(E)** The ratio of ABA/GA content in seed of WT and the *lmm9150* mutant 30 days after flowering. **(F)** The germination rate of *lmm9150* mutant and WT 30 days after flowering. Mean and SD were obtained from three replicates. Statistical analysis was performed using Student’s *t*-test, ^∗^ and ^∗∗^ indicates *P* < 0.05 and *P* < 0.01, respectively.

*OsABA2* encodes XanDH, an enzyme involves in ABA biosynthesis pathway (Endo et al., 2014). We hypothesized that mutation in this gene should influence the activity of XanDH and ABA level. To this end, we examined the activity of XanDH in leaf blades at three developmental stages. The results indicated that XanDH activity in *lmm9150* was significantly lower than that in WT at all the tested stages (**Figure 7A**). Next, we examined the ABA level in leaf blades, seeds, and stems of *lmm9150*. Consistent with the lower activity of XanDH in *lmm9150*, ABA content in leaves, seeds, and stems were all significantly lower than that in the WT at all the tested developmental stages (**Figures 7B–D**). By contrast, the GA content in leaf blades, seeds and stems were all significantly higher than that of WT (**Supplementary Figures S7A–C**). Consistently, ABA/GA ratio was dramatically decreased in leaf blades, seeds and stems, respectively, which may explain why the seeds of *lmm9150* showed lack of dormancy (**Figures 7E,F** and **Supplementary Figures S7D,E**). Taken together, we speculated that ABA deficiency likely resulted in the phenotypes of *lmm9150*.

**FIGURE 8:**
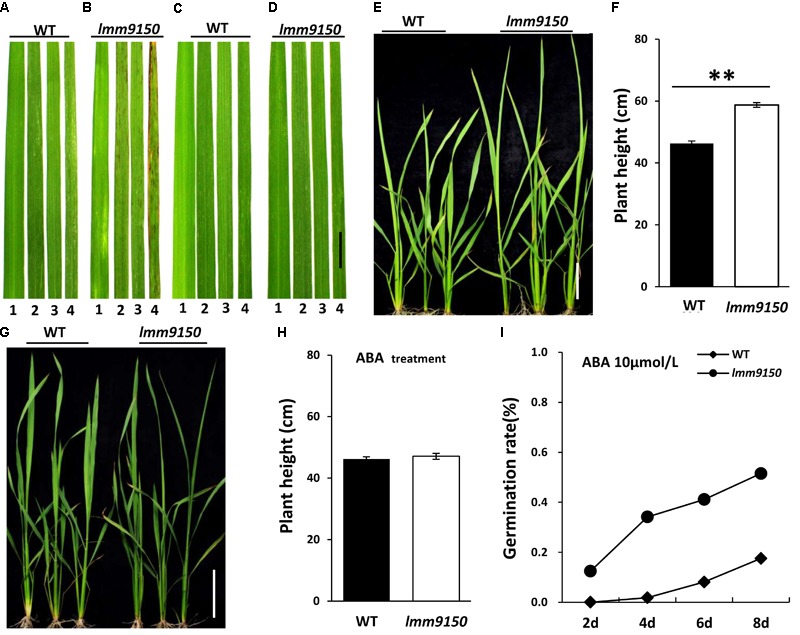
Analysis of relationship between abscisic acid (ABA) and plant development in *lmm9150* mutant. **(A–D)** Phenotypic changes in the leaf blades of wild type (WT) and *lmm9150* after ABA-treatment. The *lmm9150* shows lesion mimic spots without ABA-treatment **(A,B)** and phenotype of the *lmm*9150 mutant is similar to WT when *lmm9150* was treated with 100 μmol/L ABA for 15 days **(C,D)**. The numbers 1–4 represent the first top leaf, the second top leaf, the third top leaf, and the fourth top leaf, respectively. **(E–H)** Change of plant height of *lmm9150* mutant and WT 20 days after ABA-treatment at seedling stage. Images show that the plant height of *lmm9150* and WT after water treatment **(E,F)** and ABA-treatment **(G,H)**, respectively. The experiments were repeated twice with similar results **(A–D,E,G)**. Data were obtained from 10 seedlings **(F,H)**. **(I)** Comparison of seed germination rate in WT and *lmm9150* mutant with 10 μmol/L ABA. Mean was obtained from three replicates. Statistical analysis was performed using Student’s *t*-test, ^∗∗^ indicate *P* < 0.01.

Next, we applied ABA onto the seedlings of *lmm9150* and WT. Such ABA-treatment led to three interesting changes of phenotypes in *lmm9150*. (1) A total of 20 days after treatment, the *lmm9150* leaves in mock treatment displayed red brown spots from the older leaves (**Figures 8A,B**). In contrast, the *lmm9150* leaves in ABA-treatment grew as healthy as that of the WT leaves (**Figures 8C,D**). (2) A total of 20 days after treatment, the plant height of *lmm9150* was significantly higher than that of WT in mock treatment (**Figures 8E,F**). In contrast, the plant height of *lmm9150* after ABA-treatment was similar to that of WT at seedling stage (**Figures 8G,H**). (3) The water loss rate of *lmm9150* was obviously higher than that of WT plants at seedling stage (**Supplementary Figure S8A**). However, after ABA-treatment, the water loss rate of *lmm9150* was similar to that of WT (**Supplementary Figure S8B**).

In addition, we tested the effect of different concentration of ABA on germination of seeds in ABA-containing media. The germination rate of *lmm9150* seeds decreased when the concentration of ABA increased in a certain range, reflecting exogenous ABA could effectively inhibit seed germination (**Figure 8I** and **Supplementary Figures S8C,D**). Both the seeds of *lmm9150* and WT could not germination in media containing 100 μmol/L ABA (**Supplementary Figure S8E**), indicating that this concentration may be the lethal dosage.

Therefore, all the examined phenotypes of *lmm9150*, such as lesion mimic spots, plant height, water loss rate, and seed germination, could be rescued through the exogenous application of ABA.

## Discussion

Lesion mimic mutants are an ideal tool to investigate the association between PCD and defense responses in plants. In the present study, we isolated a rice lesion mimic mutant, *lmm9150*, from EMS-mutagenized *indica* cultivar Yixiang1B. The *lmm9150* mutant showed ABA deficiency which likely resulted in some novel and some expected phenotypes, such as spontaneous cell death, pre-harvest sprouting and enhanced growth of stem and leaf (**Figures 1, 2, 4**). Map-based cloning and CRISPR/Cas9-aided knocking-out identified a point mutation at the *LOC_Os03g59610* (*OsABA2*) locus encoding the xanthoxin dehydrogenase (XanDH) that catalyzes Xan into ABAld in the ABA biosynthesis pathway (Endo et al., 2014). Previously, *OsABA2* is reported to be able to restore the *ataba2* mutant in *Arabidopsis* (Endo et al., 2014), indicating that the function of *ABA2* in ABA biosynthesis pathway may be conserved in monocot and dicot plants.

The *lmm9150* mutant displayed spontaneous cell death on the third leaves starting from the seedling stage to the flag leaf at the yellow mature stage (**Figure 1A** and **Supplementary Figures S1A,B**), which was accompanied with excessive accumulation of H_2_O_2_ in the mutant (**Figure 2C**), similar to other rice lesion mimic mutants (Zhu et al., 2016; Liu et al., 2017; Wang et al., 2017). H_2_O_2_ is a major by-product of β-oxidation and acts as a signal molecule in the promotion of cell death (Ren et al., 2002; Zhang et al., 2003; Gechev et al., 2005). Thus, the elevated H_2_O_2_ in *lmm9150* may have contributed to the formation of lesions. It has been reported that ABA suppresses PCD through increasing activities of ROS scavenging enzymes in barley (Bethke et al., 1999; Fath et al., 2001). In maize, loss of ABA synthesis, such as the ABA-deficient mutant *viviparous9* (*vp9*), leads to the early onset of endosperm cell death (Young and Gallie, 2000). Here, we identified that *lmm9150* was an ABA-deficient mutant (**Figures 7B–D**) and exhibited cell death lesions on leaves (**Figure 1A** and **Supplementary Figures S1A,B**). Exogenous application of ABA could inhibit the formation of lesions in *lmm9150* (**Figures 8A–D**), confirming that deficiency in ABA biosynthesis can lead to formation of lesion mimics in rice. Nevertheless, the other rice ABA-deficient mutants, *osaba1, phs1, phs2, phs3*, and *phs4*, did not exhibit lesion mimic spots. H_2_O_2_ level of these mutants was not detected and the superoxide accumulation (O_2_^-^) significantly increased in *phs3* mutant. It is unknown why these ABA-deficient mutants do not exhibit lesion mimic phenotypes in rice (Agrawal et al., 2001; Fang et al., 2008).

During the lesion mimic formation, disease resistance responses are often auto-activated in LMMs, leading to enhanced resistance to pathogens in rice (Zhu et al., 2016; Liu et al., 2017; Wang et al., 2017). Consistent with this, *lmm9150* displayed enhanced resistance to both bacterial blight and rice blast diseases (**Figure 4** and **Supplementary Figure S2**), similar to the enhanced disease resistance in the other ABA-deficient mutants, such as *aba2-1, aba3-1* in *Arabidopsis* and *sit* in tomato (Xiao et al., 2017). Intriguingly, we noticed that the lesions of bacterial disease on flag leaves were longer than those on the second leaves in *lmm9150* (**Figure 4C** and **Supplementary Figure S2D**). This could be due to the earlier formation of lesions on the second leaf than that on the flag leaf, as the formation of lesions features the activation of defense against pathogens. Consistent with the enhanced resistance of *lmm9150* to pathogens, the expression of three *PR* genes, *PR1a, PR1b*, and *PR10* were significantly upregulated in *lmm9150* (**Figure 4A**). Moreover, the expression of two marker genes of JA-biosynthesis pathway was upregulated in the mutant, whereas the expression of two marker genes of SA signaling pathway was not obviously changed (**Figure 4E** and **Supplementary Figure S2F**). Consistent with this, JA level in *lmm9150* was significantly increased, but SA level was the same as the WT (**Figure 4F** and **Supplementary Figure S2G**). The hormones, including ABA, JA, and SA, are secondary signal molecules involved in defense responses (Zeevaart and Creelman, 1988; Creelman and Mullet, 1997; Dempsey et al., 1997). It has been reported that the antagonistic interactions between ABA and JA–ET signaling pathway regulate disease resistance in *Arabidopsis* (Anderson et al., 2004). However, JA signaling and SA signaling can be mutually antagonistic or synergistic in defense response (Takahashi et al., 2004; Bari and Jones, 2009; Francisco et al., 2016). Therefore, the cross-talk between JA and SA is very complicated (Proietti et al., 2013). In addition, SA signaling is usually associated with the upregulation of *PR* genes (Vallad and Goodman, 2004; Zarate et al., 2007; Jiang et al., 2009). We also detected significantly increased expression of *PR* genes in *lmm9150*, although SA level had no obvious change (**Figure 4A** and **Supplementary Figure S2G**), which could be due to the activation of defense responses by JA signaling, because JA could also activate the expression of *PR* genes (Zarate et al., 2007). Collectively, our results indicated that the mutation of *OsABA2* may alter ABA biosynthesis, which in turn, promote JA signaling pathway to activate defense against different pathogens. However, the exact mechanisms of the decreased level of ABA in *lmm9150* that enhances *PR* gene expression, JA level, and defense against pathogens are yet to be determined in the future.

Seed dormancy and germination are mainly governed by ABA and GA (Shu et al., 2016). Generally, ABA maintains seed dormancy, whereas GA releases dormancy and promotes germination (Bewley, 1997; Shu et al., 2013; Liu et al., 2014), suggesting that the ratio of ABA/GA content is a major regulator for dormancy and germination (Fang et al., 2008; Finkelstein et al., 2008). Consistent with the previous literatures, our results showed that the ABA content was decreased and the GA content was increased, which resulted in a decreased ratio of ABA/GA and pre-harvest sprouting in the seeds of *lmm9150* (**Figures 7B,E** and **Supplementary Figure S7B**). These findings were consistent with the observations in the other ABA-deficient mutants in rice that are loss of seed dormancy and exhibit pre-harvest sprouting, including *osaba1, phs1, phs2, phs3*, and *phs4* (Agrawal et al., 2001; Fang et al., 2008). On the other hand, GA is a key regulator of release dormancy and promotion germination in plants (Peng and Harberd, 2002; Ye et al., 2015; Xiong et al., 2017). The balance of ABA/GA determines seed dormancy and germination (White et al., 2000; Fang et al., 2008; Finkelstein et al., 2008). It was possible that the dysfunction of ABA biosynthesis pathway can enhance GA biosynthesis indirectly through a feedback mechanism, as geranylgeranyl pyrophosphate (GGPP) is the common precursor for ABA and GA biosynthesis (Fray et al., 1995; Rodríguezconcepción et al., 2001) and the ABA deficiency may lead to more supply of GGPP for GA production in the mutant. Therefore, it is explainable that the lower ABA/GA ratio in *lmm9150* contributed to its pre-harvest sprouting phenotype (**Figures 7C,E** and **Supplementary Figure S7B**).

In *Arabidopsis, ataba2* displays dwarfism and small size leaf (Cheng et al., 2002; Miguel et al., 2002). In contrast, we found that the plant height of *lmm9150* was higher than that of WT and the flag leaf of *lmm9150* was longer than that of WT (**Figures 1D,E**), suggesting that the function of ABA2 in regulating growth in rice is different from that in *Arabidopsis*, although both genes are involved in ABA biosynthesis. We showed that mutation in OsABA2 led to decreased level of ABA content (**Figures 7B–D**), but increased level of GA content (**Supplementary Figures S7A–C**), and exogenous application of ABA could rescue the plant height phenotype of the *lmm9150* mutant (**Figures 8E–H**), suggesting that OsABA2 negatively regulates plant height and leaf length through modulation of ABA and GA levels.

Together, our data demonstrated that novel functions of *OsABA2* in modulating plant growth, cell death, and disease resistance, in addition to the expected function in seed dormancy and add new information of the roles of ABA in different biological processes in plant.

## Author Contributions

XW designed and performed the project. WW and ML directed the research. YL, QB, PX, and TW performed the analysis of agronomic traits, histochemical staining, shading treatment, and assessed rice disease resistance. YL and QB carried out genetic analysis, map-based cloning, and germination assay. DG and YP performed the mutation analysis and sequence analysis. XD, HZ, XC, and AA assisted in the data analysis. YL, XW, and WW wrote the manuscript. All authors read and approved the final manuscript.

## Conflict of Interest Statement

The authors declare that the research was conducted in the absence of any commercial or financial relationships that could be construed as a potential conflict of interest.

## Footnotes

1 http://www.gramene.org/microsat

2 http://rise2.genomics.org.cn/page/rice/index.jsp

3 http://primer3.ut.ee/

4 http://ensembl.gramene.org/Tools/Blast?db=core

5 http://rice.plantbiology.msu.edu/

## Supplementary Material

FIGURE S1 Phenotypic characterizations of *lmm9150* mutant. **(A,B)** Comparison of four leaves between wild type (WT) and *lmm9150* mutant exhibits the lesion mimic spots of *lmm9150* at tillering stage **(A)** and mature stage **(B)**. The numbers 1, 2, 3 and 4 represent the first top leaf, the second top leaf, the third top leaf and the fourth top leaf, respectively. **(C–F)** Quantification analysis of tiller numbers **(C)**, grains setting rates **(D)**, grains weight per plant **(E)**, 1000-grains weight **(F)**, respectively. Data were obtained from 10 plants of wild type and mutant **(C–E)**. Mean of 1000-grains weight obtained from 10 replicates. Statistical analysis was performed using Student’s *t*-test, ^∗^ and ^∗∗^ indicates *P* < 0.05 and *P* < 0.01, respectively. Scale bar: 2 cm in **(A,B)**.

FIGURE S2 Determination of wild type (WT) and *lmm9150* plants resistance to bacterial blight and rice blast diseases. **(A–D)** The disease lesion leaves of the wild type (WT) and *lmm9150* mutant were inoculated with *Xanthomonas oryzae* pv. *oryzae* (*Xoo*) strains, X004, P6 and P3, at 15 days post-inoculation (dip). The image **(D)** shows that the disease lesions of WT are longer than that of *lmm9150* and the flag leaf disease lesions of *lmm9150* are longer than that of the second leaf of mutant. FL: the flag leaf; SL: the second leaf. Data were obtained from 20 leaves of main panicles. **(E)** The disease lesions of leaves of the wild type (WT) and *lmm9150* mutant were inoculated 5 days with *Magnaporthe* oryzae (*M*. oryzae) strain, Tetep. The experiments were repeated twice times with similar results. **(F)** Quantitative reverse-transcription PCR (qRT-PCR) data show the relative expression levels of salicylic acid (SA) signaling-related genes in *lmm9150* and wild type (WT). **(G)** Comparison of SA content in leaf of wild type (WT) and *lmm9150*. SS: seedling stage; TS: tillering stage. The content of hormones was tested by enzyme-linked immunosorbent assay (ELISA) method. Mean and standard deviation were obtained from three measurements **(F,G)**. The housekeeping gene *Ubiquitin5* (*Ubq5*) was used as control. Statistical analysis was performed using Student’s *t*-test, ^∗^and^∗∗^ indicate *P* < 0.05 and *P* < 0.01, respectively. Scale bar: 1 cm in **(E)**.

FIGURE S3 Comparison of the amino acid sequences of xanthoxin dehydrogenases. Box I indicates cofactor binding site and box II indicates catalytic activity site. The arrow indicates the mutation site of OsABA2 in *lmm9150* mutant. BRADI_1g04320 in Brachypodium, GRMZM2G332976 in Zea mays, LOC8057083 in Sorghum bicolor, AT1G52340.1 in *Arabidopsis*, LOC102577811 in Solanum tuberosum. These symbols describe conversation degree, “^∗^, **:** and. ” indicates strong, middle and weak respectively.

FIGURE S4 Comparison of candidate gene sequence. *LOC_Os03g59610* is the reference sequence and comes from a *japonica* rice Nipponbare. Yixiang1B-CDS sequence was cloned from an *indica* cv. Yixiang1B, which is the wild type of *lmm9150* mutant. The arrow indicates that 9-bp deletion is found in Yixiang1B.

FIGURE S5 Mutational types of 5 knockout lines. Mutational types of #1, #3, #4 and #5 knockout lines are deletion. Mutational type of #2 knockout line is insertion. The black triangles indicate the mutational sites.

FIGURE S6 Determination of wild type (WT) and a knockout line plants resistance to bacterial blight and rice blast diseases. **(A–F)** The disease lesion of leaves of the wild type (WT) and one of knockout lines were inoculated with *Xanthomonas oryzae* pv. *oryzae* (*Xoo*) strains, 8428, P3 and X004, at 15 days post-inoculation. KL: knockout lines; FL: the flag leaf; SL, the second leaf. Data were obtained from 20 leaves of main panicles. **(G)** The disease lesions of leaves of the wild type (WT) and one of knockout lines was inoculated 5 days with *Magnaporthe* oryzae (*M*. oryzae) strain, Tetep. The experiments were repeated twice times with similar results. Scale bar: 1cm in **(G)**. Statistical analysis was performed using Student’s *t*-test, ^∗∗^ indicates *P* < 0.01.

FIGURE S7 Comparison of gibberellin (GA) and ABA/GA content ratio in the wild type (WT) and *lmm9150* mutant. **(A–C)** Comparison of gibberellin (GA) content in leaves **(A)**, seeds **(B)** and stems **(C)** between the wild type (WT) and *lmm9150* mutant at different developmental stages. SS: seedling stage; TS: tillering stage; MS: mature stage; MRS: milk ripe stage; DS: dough stage; YMS: yellow mature stage. GA content was tested by enzyme-linked immunosorbent assay (ELISA) method. **(D,E)** The ratio of ABA/GA content in leaves **(D)** and stems **(E)** of the wild type (WT) and the *lmm9150* mutant. Mean and standard deviation were obtained from three measurements. Statistical analysis was performed using Student’s *t*-test, ^∗^ and ^∗∗^ indicates *P* < 0.05 and *P* < 0.01, respectively.

FIGURE S8 Effects of ABA on water loss rate and germination in *lmm9150* mutant. **(A,B)** Water loss assays for the leaves of the wild type (WT) and *lmm9150* mutant were examined at seedling stage. **(C–E)** Investigation of germination rate in *lmm9150* mutant and wild type seeds with different concentration of ABA (0.1, 1 and 100 μmol/L). Mean were obtained from three measurements.

TABLE S1 List of primers for qRT-PCR.

TABLE S2 List of polymorphic molecular markers for mapping.

TABLE S3 List of targets sequences for PCR.

TABLE S4 Segregation ratio of F2 populations.
