# Supplementary figures and images for: Mutation in Rice Abscisic Acid2 Results in Cell Death, Enhanced Disease-Resistance, Altered Seed Dormancy and Development
Source: Front Plant Sci. 2018 Mar 28;9:405. doi: 10.3389/fpls.2018.00405 (PMC5882781; doi:10.3389/fpls.2018.00405)

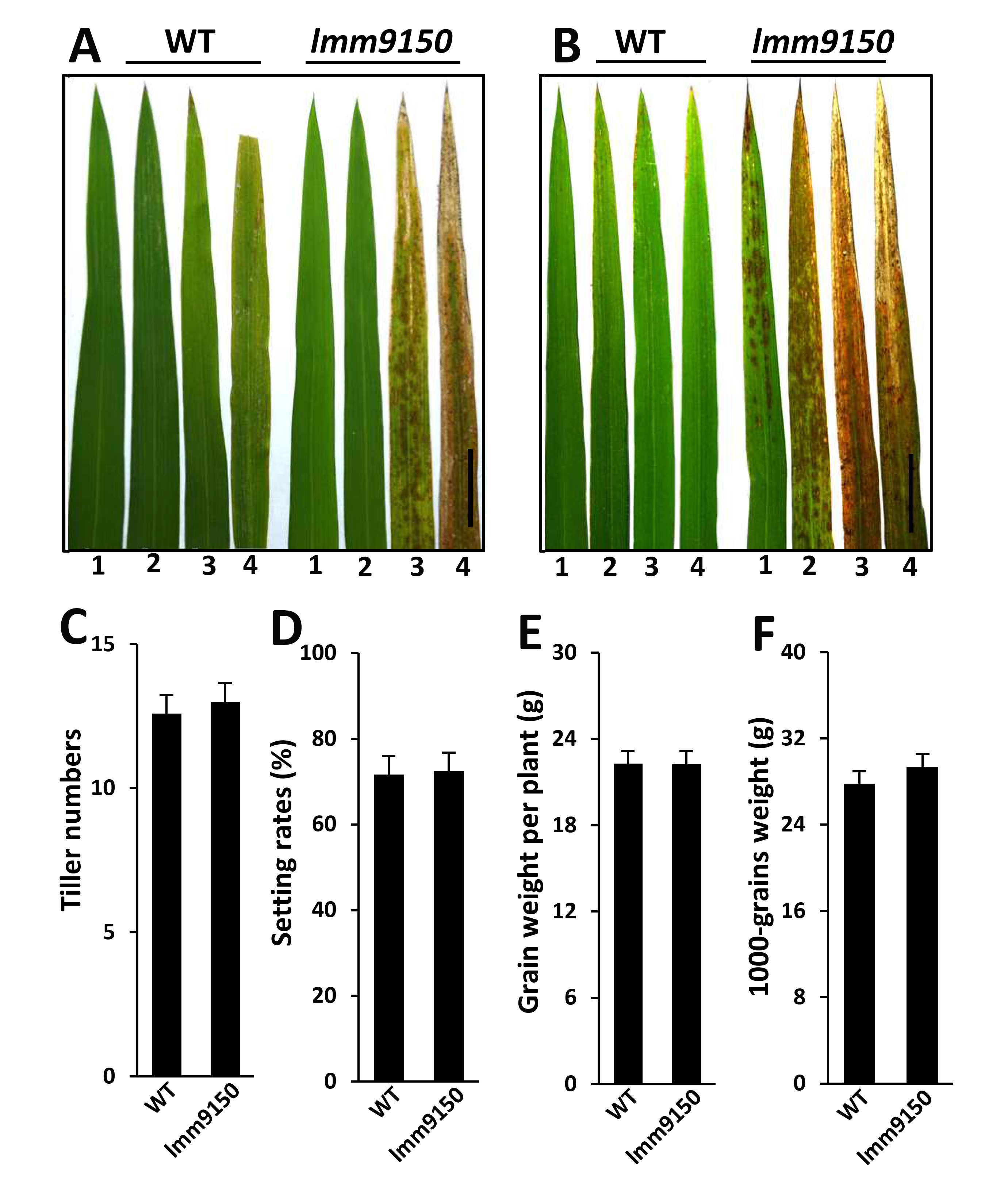

Supplement: FIGURE S1 — Phenotypic characterizations of lmm9150 mutant. (A,B) Comparison of four leaves between wild type (WT) and lmm9150 mutant exhibits the lesion mimic spots of lmm9150 at tillering stage (A) and mature stage (B). The numbers 1, 2, 3 and 4 represent the first top leaf, the second top leaf, the third top leaf and the fourth top leaf, respectively. (C–F) Quantification analysis of tiller numbers (C), grains setting rates (D), grains weight per plant (E), 1000-grains weight (F), respectively. Data were obtained from 10 plants of wild type and mutant (C–E). Mean of 1000-grains weight obtained from 10 replicates. Statistical analysis was performed using Student’s t-test, ∗ and ∗∗ indicates P < 0.05 and P < 0.01, respectively. Scale bar: 2 cm in (A,B). [file Image_1.JPEG]

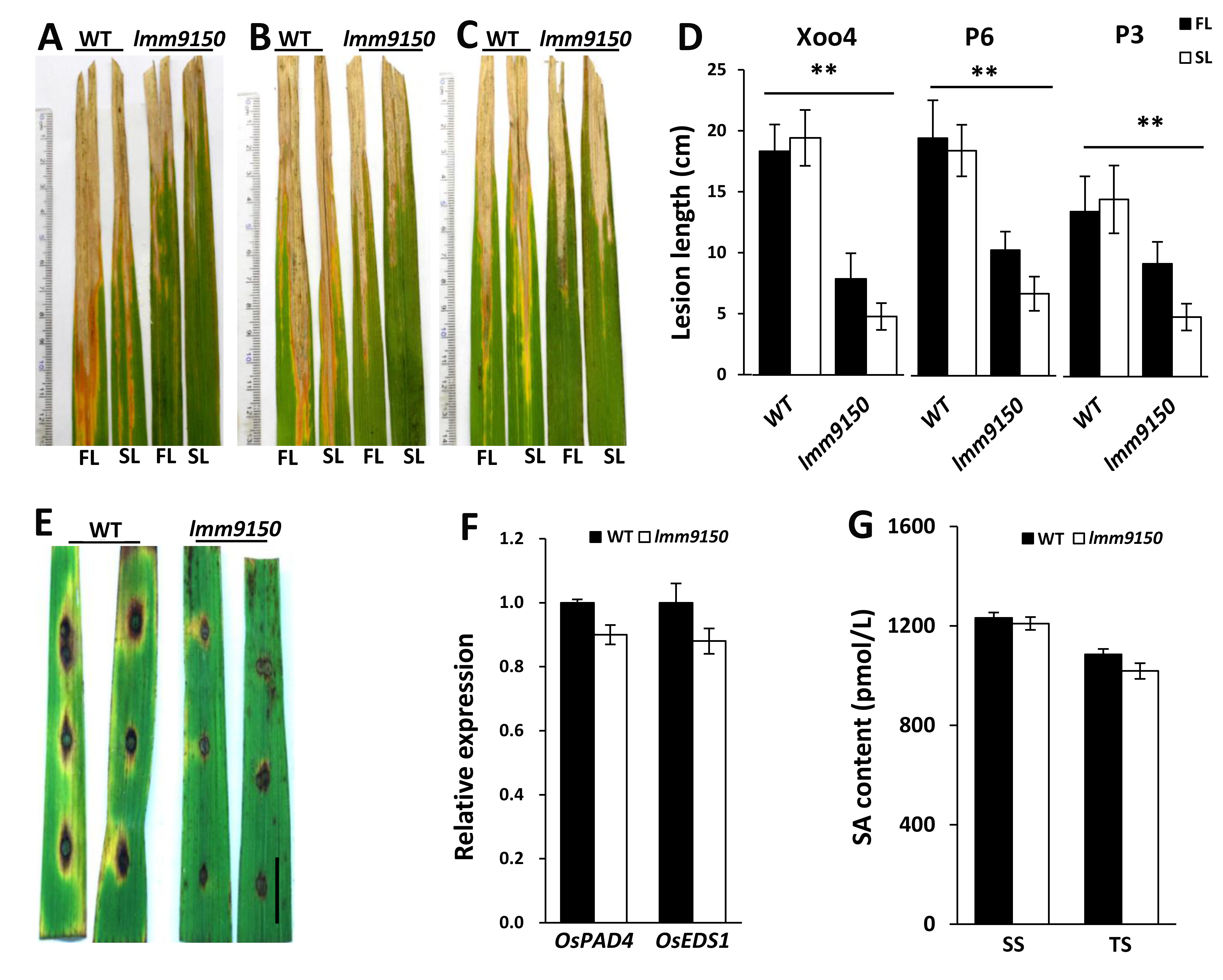

Supplement: FIGURE S2 — Determination of wild type (WT) and lmm9150 plants resistance to bacterial blight and rice blast diseases. (A–D) The disease lesion leaves of the wild type (WT) and lmm9150 mutant were inoculated with Xanthomonas oryzae pv. oryzae (Xoo) strains, X004, P6 and P3, at 15 days post-inoculation (dip). The image (D) shows that the disease lesions of WT are longer than that of lmm9150 and the flag leaf disease lesions of lmm9150 are longer than that of the second leaf of mutant. FL: the flag leaf; SL: the second leaf. Data were obtained from 20 leaves of main panicles. (E) The disease lesions of leaves of the wild type (WT) and lmm9150 mutant were inoculated 5 days with Magnaporthe oryzae (M. oryzae) strain, Tetep. The experiments were repeated twice times with similar results. (F) Quantitative reverse-transcription PCR (qRT-PCR) data show the relative expression levels of salicylic acid (SA) signaling-related genes in lmm9150 and wild type (WT). (G) Comparison of SA content in leaf of wild type (WT) and lmm9150. SS: seedling stage; TS: tillering stage. The content of hormones was tested by enzyme-linked immunosorbent assay (ELISA) method. Mean and standard deviation were obtained from three measurements (F,G). The housekeeping gene Ubiquitin5 (Ubq5) was used as control. Statistical analysis was performed using Student’s t-test, ∗and∗∗ indicate P < 0.05 and P < 0.01, respectively. Scale bar: 1 cm in (E). [file Image_2.JPEG]

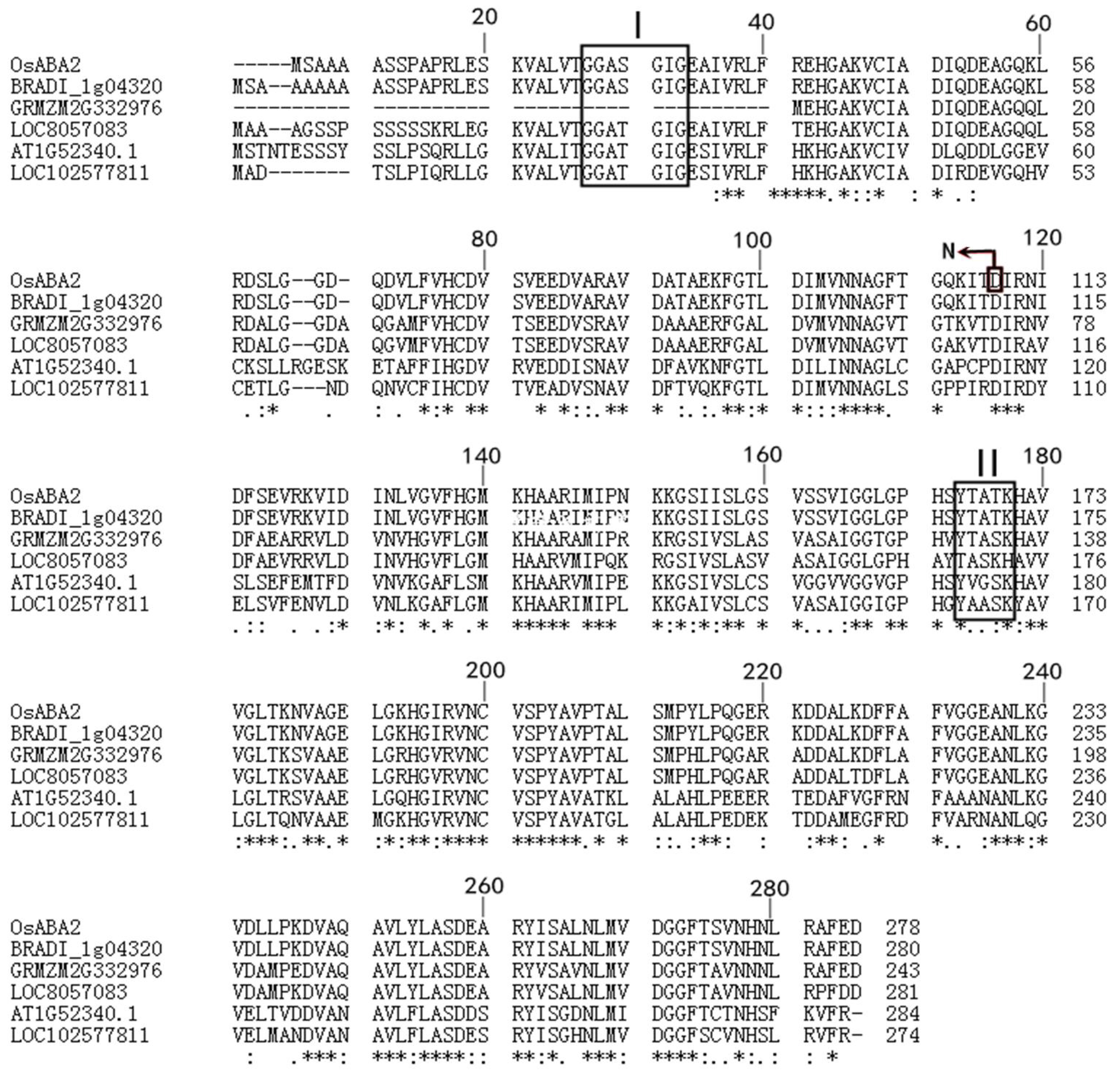

Supplement: FIGURE S3 — Comparison of the amino acid sequences of xanthoxin dehydrogenases. Box I indicates cofactor binding site and box II indicates catalytic activity site. The arrow indicates the mutation site of OsABA2 in lmm9150 mutant. BRADI_1g04320 in Brachypodium, GRMZM2G332976 in Zea mays, LOC8057083 in Sorghum bicolor, AT1G52340.1 in Arabidopsis, LOC102577811 in Solanum tuberosum. These symbols describe conversation degree, “∗, : and. ” indicates strong, middle and weak respectively. [file Image_3.JPEG]

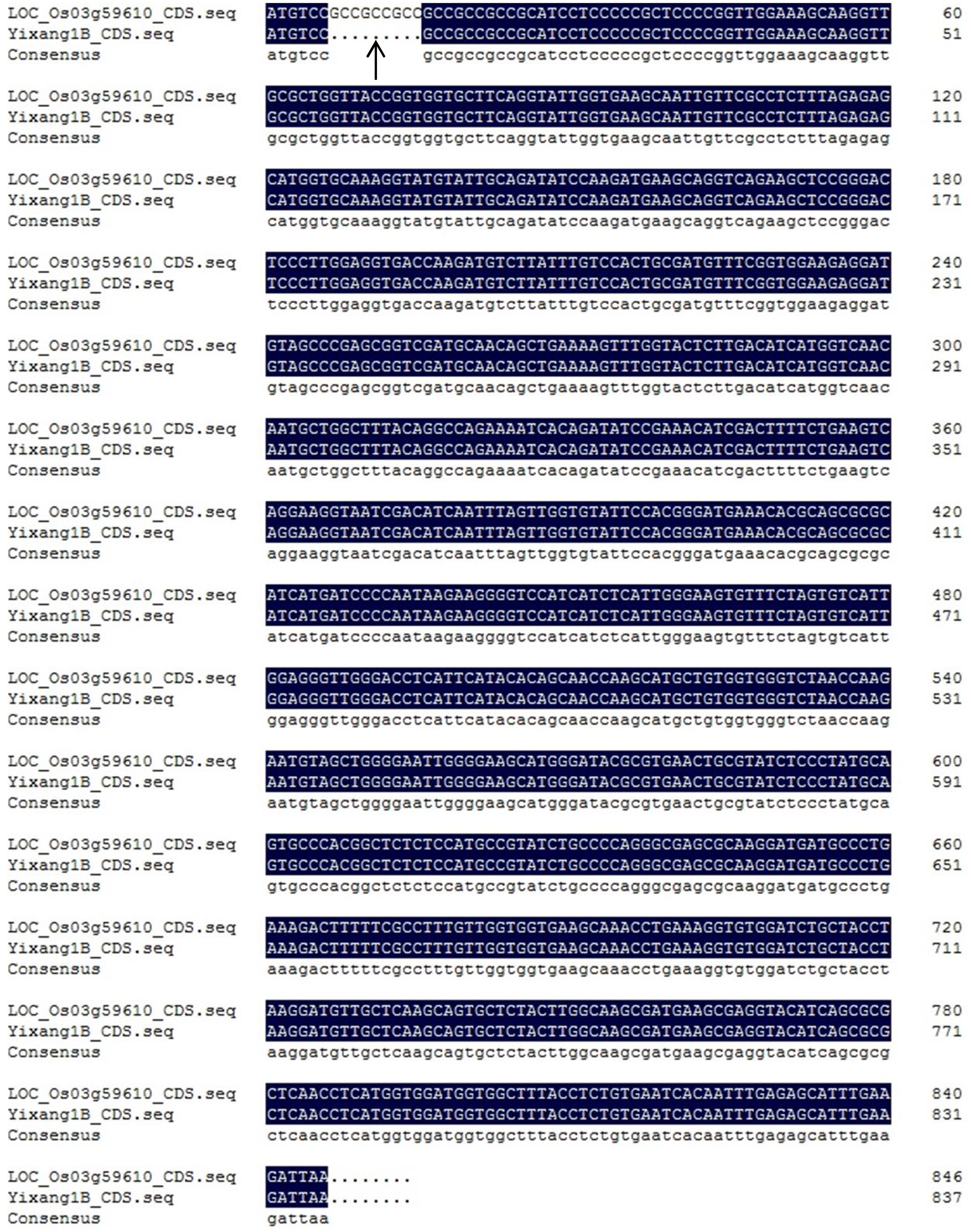

Supplement: FIGURE S4 — Comparison of candidate gene sequence. LOC_Os03g59610 is the reference sequence and comes from a japonica rice Nipponbare. Yixiang1B-CDS sequence was cloned from an indica cv. Yixiang1B, which is the wild type of lmm9150 mutant. The arrow indicates that 9-bp deletion is found in Yixiang1B. [file Image_4.JPEG]

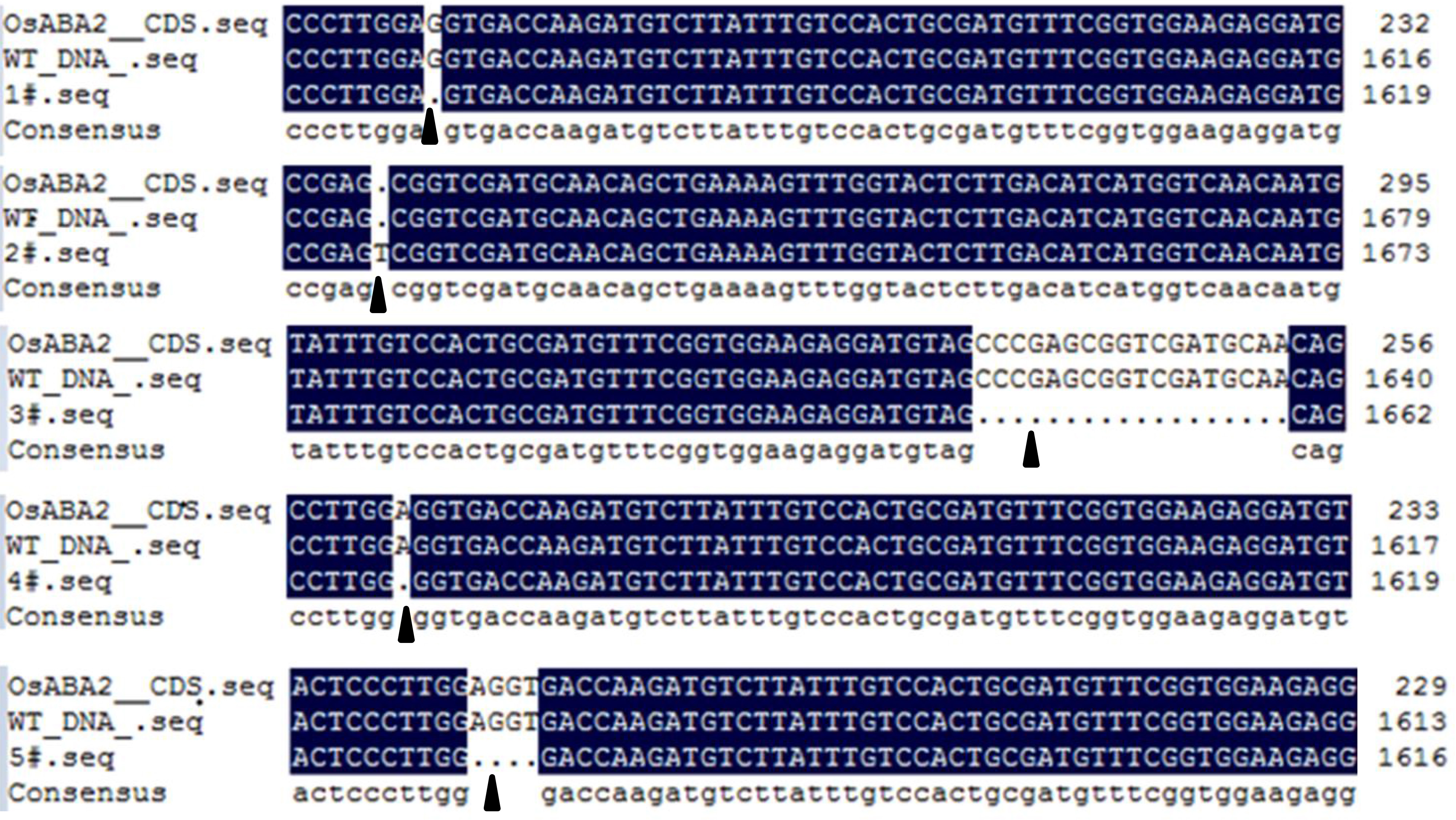

Supplement: FIGURE S5 — Mutational types of 5 knockout lines. Mutational types of #1, #3, #4 and #5 knockout lines are deletion. Mutational type of #2 knockout line is insertion. The black triangles indicate the mutational sites. [file Image_5.JPEG]

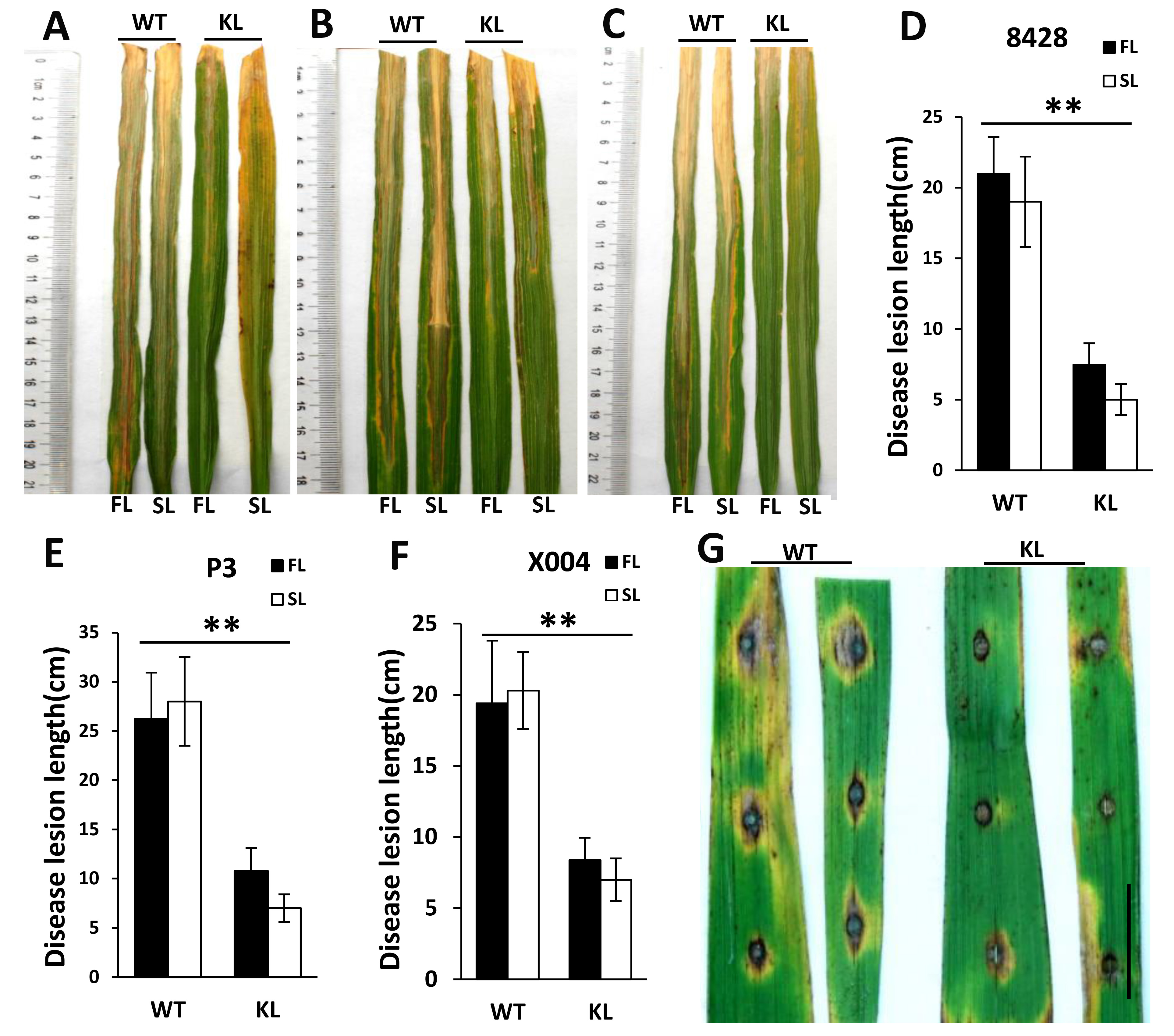

Supplement: FIGURE S6 — Determination of wild type (WT) and a knockout line plants resistance to bacterial blight and rice blast diseases. (A–F) The disease lesion of leaves of the wild type (WT) and one of knockout lines were inoculated with Xanthomonas oryzae pv. oryzae (Xoo) strains, 8428, P3 and X004, at 15 days post-inoculation. KL: knockout lines; FL: the flag leaf; SL, the second leaf. Data were obtained from 20 leaves of main panicles. (G) The disease lesions of leaves of the wild type (WT) and one of knockout lines was inoculated 5 days with Magnaporthe oryzae (M. oryzae) strain, Tetep. The experiments were repeated twice times with similar results. Scale bar: 1cm in (G). Statistical analysis was performed using Student’s t-test, ∗∗ indicates P < 0.01. [file Image_6.JPEG]

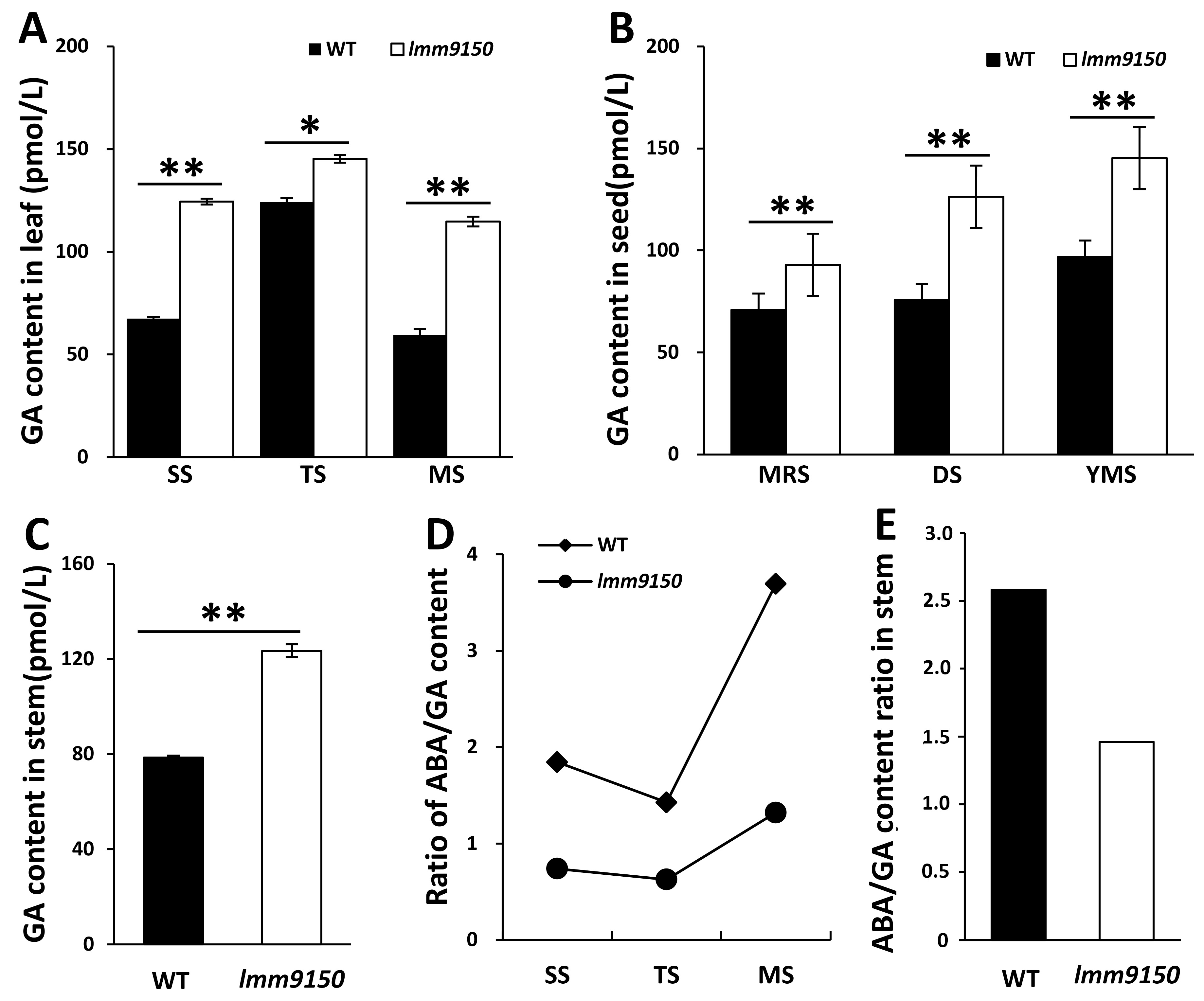

Supplement: FIGURE S7 — Comparison of gibberellin (GA) and ABA/GA content ratio in the wild type (WT) and lmm9150 mutant. (A–C) Comparison of gibberellin (GA) content in leaves (A), seeds (B) and stems (C) between the wild type (WT) and lmm9150 mutant at different developmental stages. SS: seedling stage; TS: tillering stage; MS: mature stage; MRS: milk ripe stage; DS: dough stage; YMS: yellow mature stage. GA content was tested by enzyme-linked immunosorbent assay (ELISA) method. (D,E) The ratio of ABA/GA content in leaves (D) and stems (E) of the wild type (WT) and the lmm9150 mutant. Mean and standard deviation were obtained from three measurements. Statistical analysis was performed using Student’s t-test, ∗ and ∗∗ indicates P < 0.05 and P < 0.01, respectively. [file Image_7.JPEG]

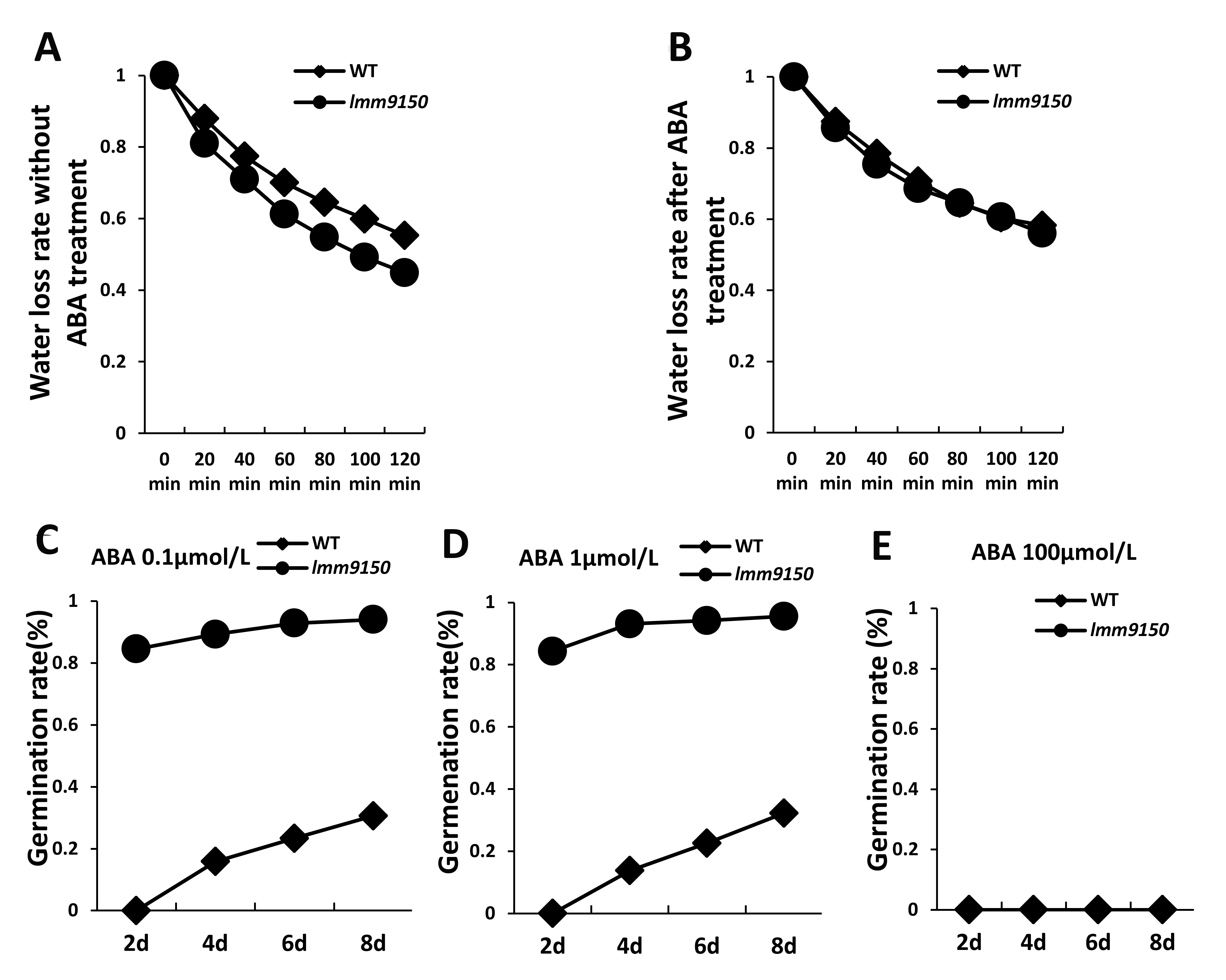

Supplement: FIGURE S8 — Effects of ABA on water loss rate and germination in lmm9150 mutant. (A,B) Water loss assays for the leaves of the wild type (WT) and lmm9150 mutant were examined at seedling stage. (C–E) Investigation of germination rate in lmm9150 mutant and wild type seeds with different concentration of ABA (0.1, 1 and 100 μmol/L). Mean were obtained from three measurements. [file Image_8.JPEG]
