# Supplementary material for: Mutation in Rice Abscisic Acid2 Results in Cell Death, Enhanced Disease-Resistance, Altered Seed Dormancy and Development
Source: Front Plant Sci. 2018 Mar 28;9:405. doi: 10.3389/fpls.2018.00405 (PMC5882781; doi:10.3389/fpls.2018.00405)
Supplement: TABLE S1 — List of primers for qRT-PCR. [file Table_1.DOCX]

**TABLE S1 List of primers for qRT-PCR**

| **Markers** | **Forward (5’-3’)** | **Reverse (5’-3’)** |
| --- | --- | --- |
| *OsCATC* | CATCCAGACCATCGACCCC | TGAGCACCATCCTCCCCAC |
| *PR1a* | CGTGTCGGCGTGGGTGT | GGCGAGTAGTTGCAGGTGATG |
| *PR1b* | TACGCCAGCCAGAGGAGC | GCCGAACCCCAGAAGAGG |
| *nPR10* | AAGTTGCAGGTGGGGGATA | CTCAATGGCGTCGATGAAG |
| *OsLOX* | GATGGCGGTGCTCGACGTGCT | GCACCTGTTCTTGAGCTTTCTAT |
| *OsAOS* | CTCGTCGGAAGGCTGTTGCT | ACGATTGACGGCGGAGGTT |
| *OsPAD4* | CCAACATGTACCGCATCAAG | GGTTGTTTCGGTGGTAGTGG |
| *OsEDS1* | CATTCCAAGAACGAGGACACTG | CAAGACTCAAGGCTAGAACCGA |
| OsGA20ox2 | GGAGCACCGCGTGATCGTGAA | CCTCAAGCTGTGCCTTGCCCTT |
| Ubq5 | AACCAGCTGAGGCCCAAGA | ACGATTGATTTAACCAGTCCATGA |
