# Supplementary material for: Mutation in Rice Abscisic Acid2 Results in Cell Death, Enhanced Disease-Resistance, Altered Seed Dormancy and Development
Source: Front Plant Sci. 2018 Mar 28;9:405. doi: 10.3389/fpls.2018.00405 (PMC5882781; doi:10.3389/fpls.2018.00405)
Supplement: TABLE S2 — List of polymorphic molecular markers for mapping. [file Table_2.DOCX]

**TABLE S2 List of polymorphic molecular markers for mapping.**

| **Markers** | **Forward (5’-3’)** | **Reverse (5’-3’)** |
| --- | --- | --- |
| I403.1 | GATAACGTGGAGGAGTCGT | TGCACTCAAAATTTTCCTCT |
| I403.4 | CAGAAGAAATTGTCTCCCC | GTTAAACACCTCTCGCAAAC |
| I403.7 | TTCAACCTGCATCCGCTC | CCATCCAAATCAGCAACAGC |
| RM143 | GTCCCGAACCCTAGCCCGAGGG | AGAGGGCCCTCCACATGGCGACC |
| I403.3 | TCTGCTCGTAGCAACAATAA | TACATTGTGAAACGGATGAA |
| I403.2 | GGCTATGCACAGTACTACGTT | GCTACACTTATTTTGGGACG |
| RM3684 | TATTTCACCTTCCTGCCACG | GAATGAGGTGGAGGATCGAC |
| RM7389 | AGCGACGGATGCATGATC | TTGAGCCGGAGGTAGTCTTG |
