# Supplementary material for: Mutation in Rice Abscisic Acid2 Results in Cell Death, Enhanced Disease-Resistance, Altered Seed Dormancy and Development
Source: Front Plant Sci. 2018 Mar 28;9:405. doi: 10.3389/fpls.2018.00405 (PMC5882781; doi:10.3389/fpls.2018.00405)
Supplement: TABLE S3 — List of targets sequences for PCR. [file Table_3.DOCX]

**TABLE S3 List of targets sequences for PCR.**

| **Name** | **Forward (5’-3’)** | **Reverse (5’-3’)** |
| --- | --- | --- |
| lmm9150-Y | CAGTGGTCTCAGGCAGACATCTTGGTCACCTCCA | CAGTGGTCTCAAAACTGGAGGTGACCAAGATGTC |
| lmm9150-B | CAGTGGTCTCAGGCAGCTGTTGCATCGACCGCTC | CAGTGGTCTCAAAACGAGCGGTCGATGCAACAGC |
| Sequencing primer | CCCAGCCTGAGATTCCGTAT | TGATTGTCCTTAAGCACCGG |
