# Supplementary material for: Mutation in Rice Abscisic Acid2 Results in Cell Death, Enhanced Disease-Resistance, Altered Seed Dormancy and Development
Source: Front Plant Sci. 2018 Mar 28;9:405. doi: 10.3389/fpls.2018.00405 (PMC5882781; doi:10.3389/fpls.2018.00405)
Supplement: TABLE S4 — Segregation ratio of F2 populations. [file Table_4.DOCX]

**TABLE S4 Segregation ratio of F_2_ populations.**

| **Cross** | **NP** | **LP** | **Segregation ratio** | **χ2 0.05<3.84** | ***P*** |
| --- | --- | --- | --- | --- | --- |
| Yixiang1B/*lmm9150* | 484 | 164 | 2.95 | 0.59 | *P*<0.05 |
| *lmm9150*/ Yixiang1B | 416 | 143 | 2.91 | 1.00 | *P*<0.05 |

Note: NP, normal phenotype plants. LP, lesion mimic phenotype plants.
